# Supplementary material for: Transient turbid water mass reduces temperature-induced coral bleaching and mortality in Barbados
Source: PeerJ. 2016 Jun 14;4:e2118. doi: 10.7717/peerj.2118 (PMC4911954; doi:10.7717/peerj.2118)
Supplement: Supplemental Information 1 [file peerj-04-2118-s001.docx]

Supplementary Information

**Transient turbid water mass reduces temperature-induced coral bleaching and mortality in Barbados**

**Hazel A. Oxenford^1^* and Henri Vallès^2^**

***^1^Centre for Resource Management and Environmental Studies, University of the West Indies, Cave Hill, Barbados***

***^2^Department of Biological and Chemical Sciences, University of the West Indies, Cave Hill, Barbados***

| a) **STT**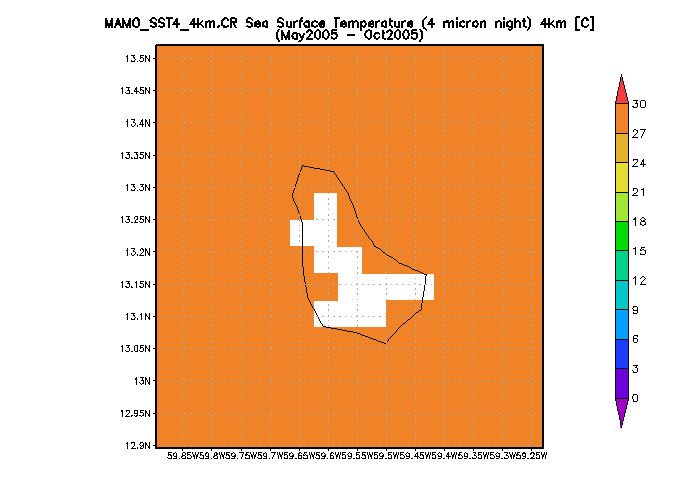 | 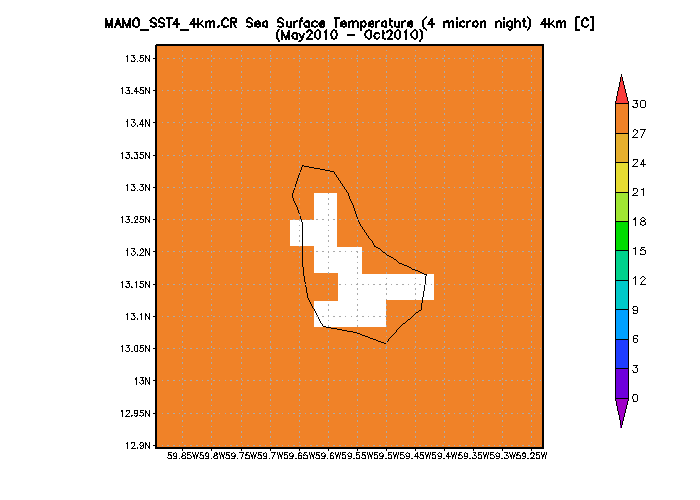 |
| --- | --- |
| b) **CDOM**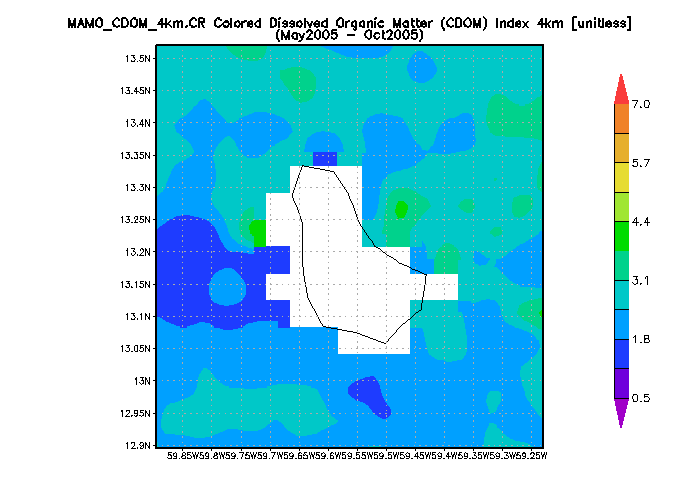 | 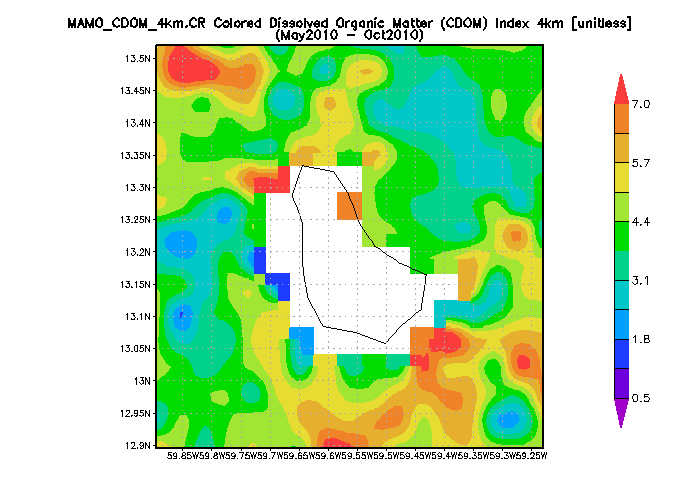 |
| c) **POC**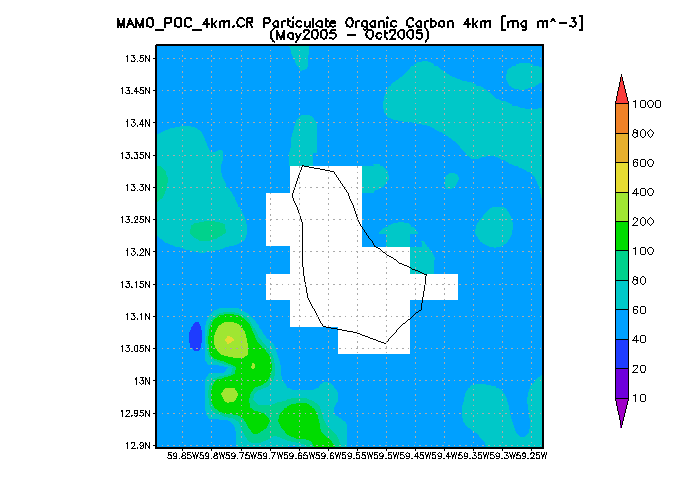 | 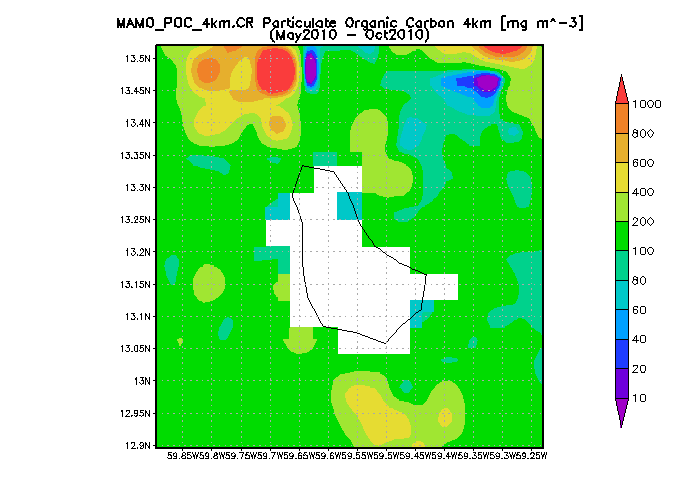 |
| d) **Chl-*a***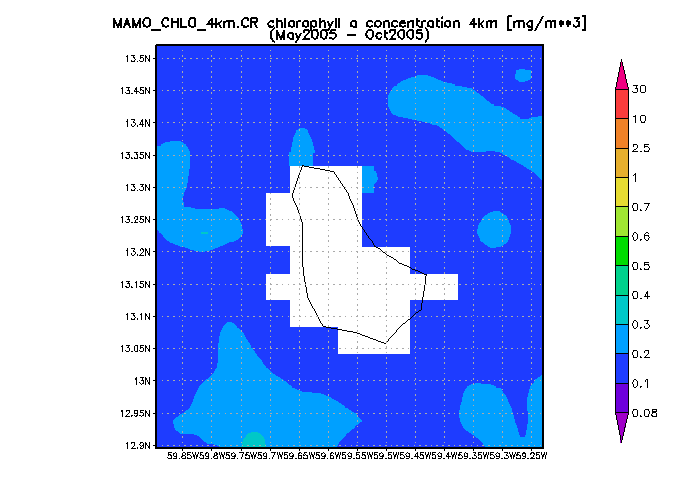 | 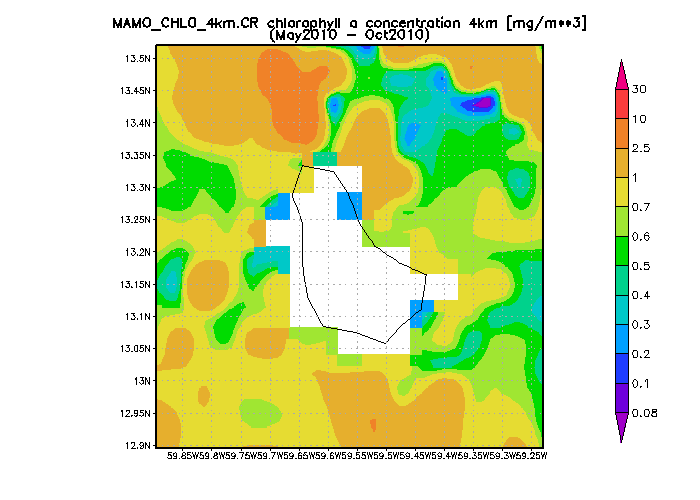 |

Figure S1 – Time-averaged satellite-imagery values for a) sea surface temperature (degrees Celsius), b) particulate organic matter (mg per m^3^), c) chlorophyll-*a* concentration (mg per m^3^) and d) coloured dissolved organic matter (unitless index) around Barbados over the May to October period in 2005 (left panels) and 2010 (right panels). Maps were created using the custom visualisation tools and data available at: <http://gdata1.sci.gsfc.nasa.gov/daac-bin/G3/gui.cgi?instance_id=ocean_month>

| a) **SST**  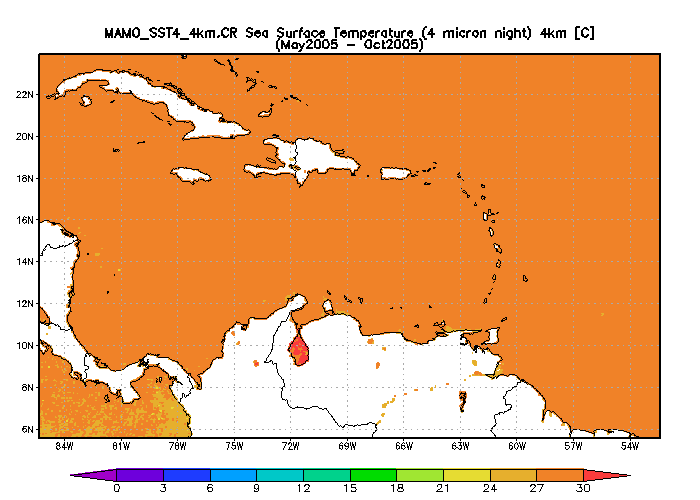 | 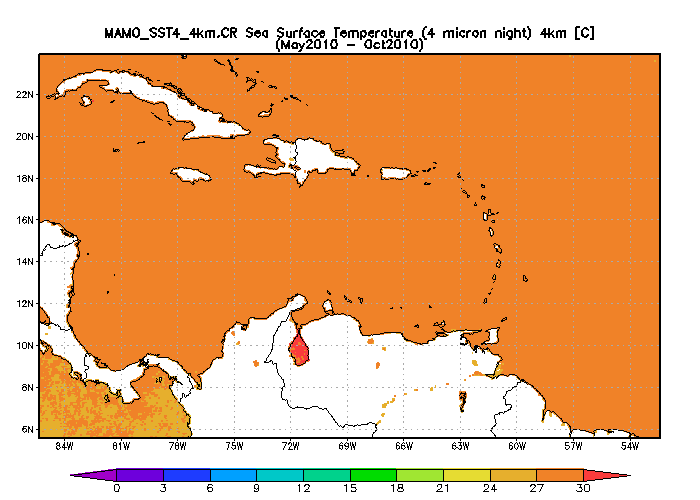 |
| --- | --- |
| b) **CDOM**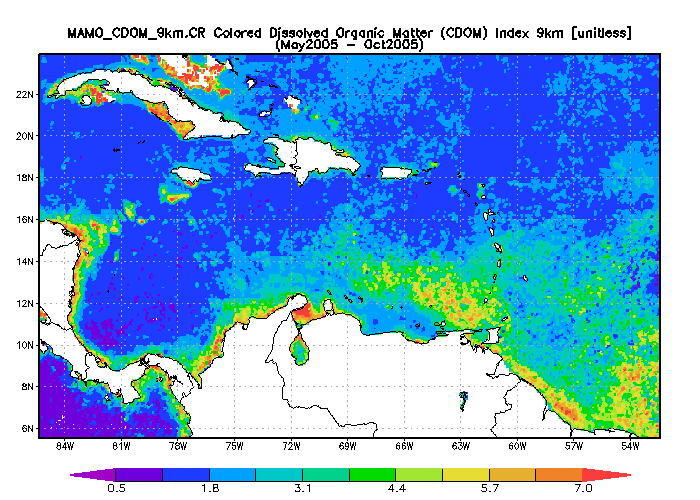 | 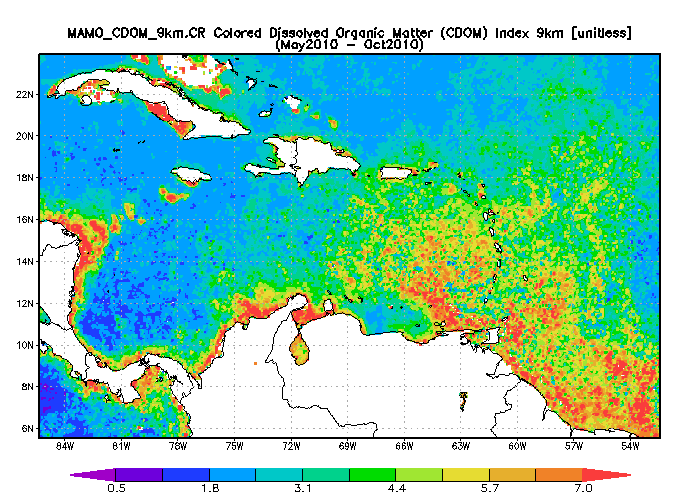 |
| c) **POC**  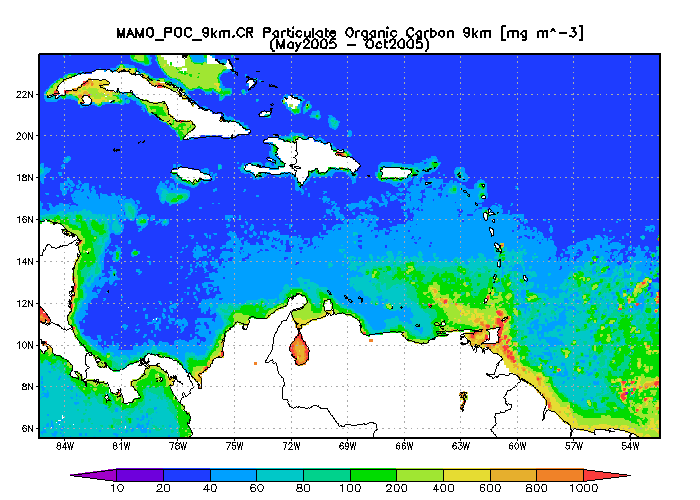 | 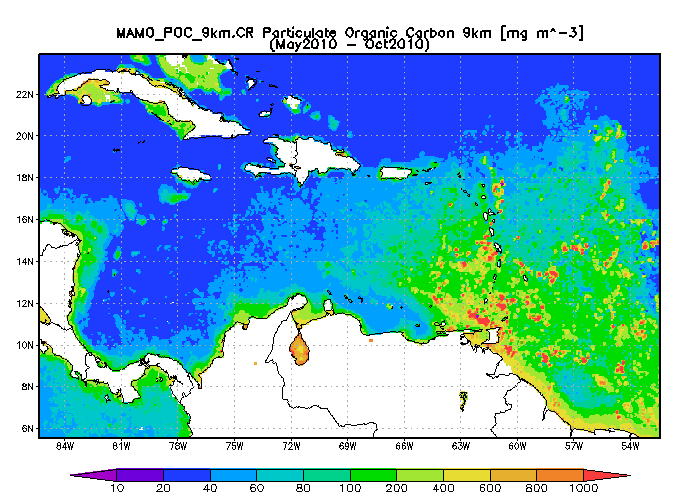 |
| d) **Chl-*a***  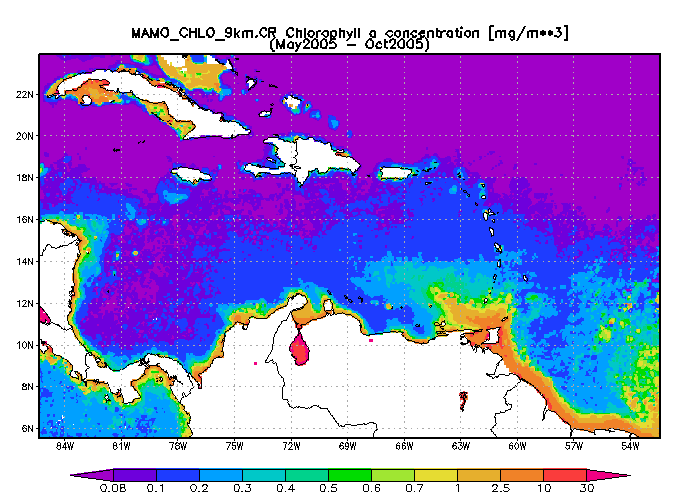 | 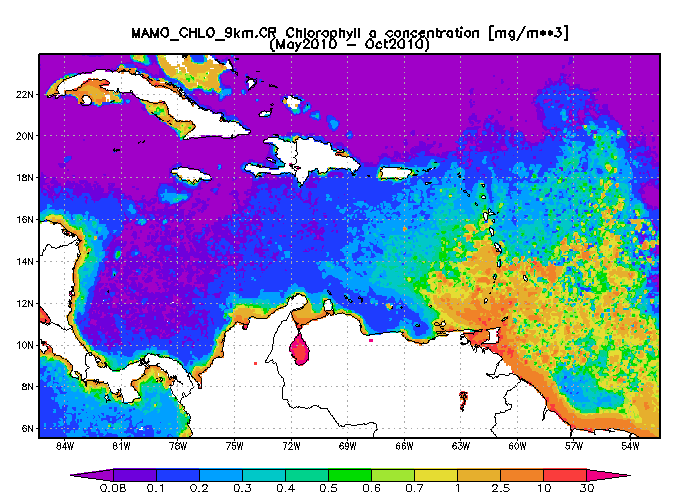 |

Figure S2 – Time-averaged satellite-imagery values for a) sea surface temperature (degrees Celsius), b) coloured dissolved organic matter (unitless index), c) particulate organic matter (mg per m^3^), and d) chlorophyll-*a* concentration (mg per m^3^) in the Wider Caribbean over the May to October period in 2005 (left panels) and 2010 (right panels). Maps were created using the custom visualisation tools and data available at: <http://gdata1.sci.gsfc.nasa.gov/daac-bin/G3/gui.cgi?instance_id=ocean_month>

Figure S3 – Daily current velocity values at a site on the (a) south coast and (b) west coast during 2005 and 2010. Missing data are shown as gaps in the time series. A 13-day period in August for which data were available across all four time series is demarcated by the two vertical lines.

Table S1 - Non-comprehensive list of publications documenting the effect of transient water masses of South American riverine origin on the hydrology of Barbados through *in situ* observations.

Borstad, G.A. 1979. ASTP at Barbados: Mesoscale Pools of Amazon River Water in the Western Tropical Atlantic. In: F. El-Baz and D. M. Warner, editors, Apollo-Soyuz Test Project. Summary Science Report. Volume II. Earth Observations and Photography. NASA, Washington. p. 481-498.

Borstad, G.A. 1982. The influence of the meandering Guiana Current and Amazon River discharge on surface salinity near Barbados. Journal of Marine Research 40: 421-434.

Borstad, G.A. 1982. The influence of the meandering Guiana Current on surface conditions near Barbados - temporal variations of *Trichodesmium* (Cyanophyta) and other plankton. Journal of Marine Research 40: 435-451.

Bowman, M.J., K.L. Stansfield, S.J. Fauria and T.C. Wilson. 1994. Coastal ocean circulation near Barbados, W. I. Spring 1990 and 1991. J Geophys Res 99-C8: 16131-16142.

Cowen, R. and L.R. Castro. 1994. Relation of coral reef fish larval distributions to island scale circulation around Barbados, West Indies. Bull Mar Sci 54: 228-244.

Cowen, R.K., S. Sponaugle, C.B. Paris, K.M.M. Lwiza, J.L. Fortuna and S. Dorsey. 2003. Impact of North Brazil Current rings on local circulation and coral reef fish recruitment to Barbados, West Indies. In: G. J. Goni and P. M. Rizzoli, editors, Interhemispheric water exchange in the Atlantic Ocean. Elsevier, Amsterdam. p. 443-462.

Kelly, P.S., K.M.M. Lwiza, R.K. Cowen and G.J. Goni. 2000. Low-salinity pools at Barbados, West Indies: Their origin, frequency, and variability. J Geophys Res 105(C8): 19699-19708.

Kidd, R. and F. Sander. 1979. Influence of Amazon River discharge on the marine production system off Barbados, West Indies. Journal of Marine Research 37: 669-681.

Kidd, R.J. 1978. The influence of Amazon River discharge and the "island mass effect" upon distribution, species diversity and numbers of zooplankton near Barbados, West Indies. McGill University, Montreal.

Paris, C.B., R.K. Cowen, K.M.M. Lwiza, D.P. Wang and D.B. Olson. 2002. Multivariate objective analysis of the coastal circulation of Barbados, West Indies: implication for larval transport. Deep-Sea Research Part I-Oceanographic Research Papers 49: 1363-1386.

Sander, F. 1981. A Preliminary Assessment of the Main Causative Mechanisms of the Island Mass Effect of Barbados. Mar Biol 64: 199-205.

Sander, F. and D.M. Steven. 1973. Organic productivity of inshore and offshore waters of Barbados: a study of the island mass effect. Bull Mar Sci 23: 771-792.

Stansfield, K.L., M.J. Bowman, S. J Fauria and T.C. Wilson. 1995. Water mass and coastal current variability near Barbados, West Indies. J Geophys Res 100: 24819-24830.

Steven, D.M. and A.L. Brooks. 1972. Identification of Amazon River water at Barbados, W. Indies, by salinity and silicate measurements. Mar Biol 14: 345-348.
